# Pantothenate Auxotrophy in a Naturally Occurring Biocontrol Yeast

**DOI:** 10.1128/aem.00884-23

**Published:** 2023-07-05

**Authors:** Maria Paula Rueda-Mejia, Andreas Bühlmann, Raúl A. Ortiz-Merino, Stefanie Lutz, Christian H. Ahrens, Markus Künzler, Florian M. Freimoser

**Affiliations:** a Agroscope, Research Division Plant Protection, Nyon, Switzerland; b Agroscope, Research Division Food Microbial Systems, Wädenswil, Switzerland; c Conway Institute, University College Dublin, Dublin, Ireland; d Agroscope, Competence Division Method Development and Analytics, Zürich, Switzerland; e SIB, Swiss Institute of Bioinformatics, Zürich, Switzerland; f Institute of Microbiology, Department of Biology, ETH Zürich, Zürich, Switzerland; Chalmers University of Technology

**Keywords:** antagonism, genome evolution, *Hanseniaspora*, transporter, vitamin, yeast

## Abstract

The genus *Hanseniaspora* is characterized by some of the smallest genomes among budding yeasts. These fungi are primarily found on plant surfaces and in fermented products and represent promising biocontrol agents against notorious fungal plant pathogens. In this work, we identify pantothenate auxotrophy of a Hanseniaspora meyeri isolate that shows strong antagonism against the plant pathogen Fusarium oxysporum. Furthermore, strong biocontrol activity *in vitro* required both pantothenate and biotin in the growth medium. We show that the *H. meyeri* isolate APC 12.1 can obtain the vitamin from plants and other fungi. The underlying reason for the auxotrophy is the lack of two key pantothenate biosynthesis genes, but six genes encoding putative pantothenate transporters are present in the genome. By constructing and using a Saccharomyces cerevisiae reporter strain, we identified one *Hanseniaspora* transporter that conferred pantothenate uptake activity to S. cerevisiae. Pantothenate auxotrophy is rare and has been described in only a few bacteria and in S. cerevisiae strains that were isolated from sake. Such auxotrophic strains may seem an unexpected and unlikely choice as potential biocontrol agents, but they may be particularly competitive in their ecological niche and their specific growth requirements are an inherent biocontainment strategy preventing uncontrolled growth in the environment. Auxotrophic strains, such as the *H. meyeri* isolate APC 12.1, may thus represent a promising strategy for developing biocontrol agents that will be easier to register than prototrophic strains, which are normally used for such applications.

**IMPORTANCE** As a precursor of the essential coenzyme A (CoA), pantothenate is present in all organisms. Plants, bacteria, and fungi are known to synthesize this vitamin, while animals must obtain it through their diet. Pantothenate auxotrophy has not been described in naturally occurring, environmental fungi and is an unexpected property for an antagonistic yeast. Here, we report that yeasts from the genus *Hanseniaspora* lack key enzymes for pantothenate biosynthesis and identify a transporter responsible for the acquisition of pantothenate from the environment. *Hanseniaspora* isolates are strong antagonists of fungal plant pathogens. Their pantothenate auxotrophy is a natural biocontainment feature that could make such isolates interesting candidates for new biocontrol approaches and allow easier registration as plant protection agents than prototrophic strains.

## INTRODUCTION

The genus *Hanseniaspora*, the teleomorph of *Kloeckera*, comprises widely distributed apiculate yeasts that are among the most abundant fungi on fruits and reach high cell densities during the early stages of fermentation in wine must (1–3). Several *Hanseniaspora* strains show potential as biocontrol agents against plant pathogens of important fruit crops. Antagonistic activity against crop spoilage molds, such as *Botrytis*, *Corynespora*, *Rhizopus*, *Penicillium*, or *Phytophthora*, has been described in grape, apple, citrus, and strawberry fruits (4–8).

In general, competition for nutrients and space, including biofilm formation, is considered the most important mechanism by which biocontrol yeasts inhibit plant-pathogenic fungi (9). Growth assays with *Hanseniaspora* culture filtrates, supernatants, and sterilized solutions did not result in mold inhibition (10). Therefore, the mechanism for antagonism was presumed to be competition for nutrients and space. However, the interest in a mechanistic understanding of biocontrol activities has grown recently and other possible mechanisms may be involved in the *Hanseniaspora* activity against plant pathogens. Inhibition of Botrytis cinerea spore germination and mycelium growth by Hanseniaspora uvarum has been described (5). Volatile and soluble compounds were also found to be involved in the biocontrol activity of *Hanseniaspora.* Specifically, volatile organic compounds (VOCs), such as 1,3,5,7-cyclooctatetraene, are produced by *H. uvarum* in the presence of *B. cinerea* on strawberry surfaces, and compounds extracted from Hanseniaspora osmophila had significant inhibitory effects on the same pathogen (11, 12).

The genus *Hanseniaspora* displays some of the smallest genomes and sets of annotated genes among the budding yeasts in the order *Saccharomycetales* (on average, 9.71 ± 1.32 Mbp and 4,708 ± 634 genes) (13). The *Hanseniaspora* clade has lost a large number of genes encoding proteins with functions in primary metabolism, cell cycle regulation, and the maintenance of genome integrity. Due to such gene losses, many *Hanseniaspora* strains are unable to metabolize certain sugars (e.g., galactose, maltose, raffinose, or melezitose), are auxotrophic for thiamine, have a disrupted methionine salvage pathway, and exhibit accelerated evolution due to their high mutation rate and genome instability (13). Considering their small genomes and limited metabolic capabilities, the success in various environmental habitats and strong competitive activity against pathogenic fungi *in vitro* are remarkable and raise the question about the underlying mechanisms.

Previously, we reported a *Hanseniaspora meyeri* isolate from apple flowers that showed strong antagonistic activity against a wide variety of important plant pathogens in agar-based competition assays (14). Here, we show that this isolate is a pantothenate auxotroph and can obtain this essential vitamin from plant roots and filamentous fungi. While the *Hanseniaspora* strain APC 12.1 (as well as other strains of this genus for which whole-genome sequences are available) lacks two genes for pantothenate biosynthesis, its genome contains six candidate pantothenate transporter genes (based on KEGG annotations). One of these transporters was shown to complement the phenotype of a Saccharomyces cerevisiae strain lacking its endogenous pantothenate symporter Fen2, encoded by a single copy gene.

In summary, these studies describe an antagonistic, environmental yeast with a reduced genome that lacks the ability to synthesize pantothenate but that can obtain this vitamin from plants and fungi in its ecological niche via at least one pantothenate transporter. Such strains might be interesting for biocontrol applications because the pantothenate auxotrophy prevents uncontrolled spread in the field but can easily be overcome by adding the vitamin to the formulation.

## RESULTS

### On plant growth medium, *H. meyeri* (APC 12.1) must obtain an essential nutrient from plant roots or other fungi.

In the past, we have identified several yeasts that strongly inhibit filamentous fungi in binary competition assays *in vitro* (14). Here, the goal was to assess yeast biocontrol activity in the presence of a host plant. The assay comprised the yeast *H. meyeri* (isolate APC 12.1), the plant pathogen Fusarium oxysporum f. sp. *lycopersici*, and tomato seedlings and was performed on plant culture medium lacking a carbon source (to prevent overgrowth by the yeast and F. oxysporum).

**FIG 1:**
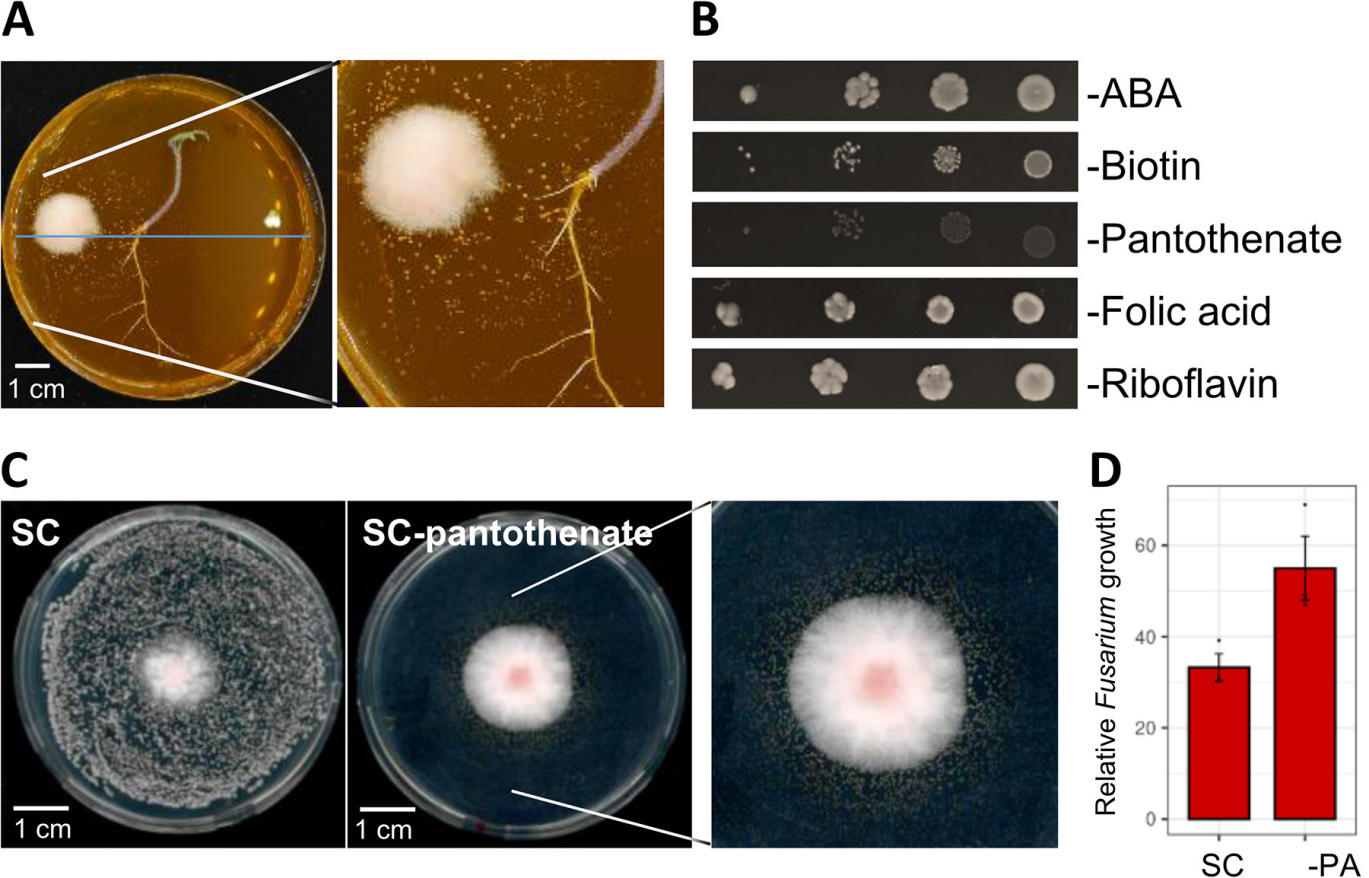
*Hanseniaspora meyeri* (APC 12.1) is auxotrophic for pantothenate. (A) Tomato-F. oxysporum-yeast interaction on MS medium. (B) *H. meyeri* (APC 12.1) growth on SC medium lacking amino benzoic acid (ABA), biotin, calcium pantothenate, folic acid, or riboflavin. *H. meyeri* was unable to grow in the absence of calcium pantothenate. The lack of either biotin or folic acid resulted in reduced growth and colony size. Medium without riboflavin or ABA permitted normal growth. (C) Fusarium*-Hanseniaspora* competition in SC and SC without pantothenate. On SC medium lacking calcium pantothenate, *H. meyeri* grew only around the F. oxysporum colony. (D) The relative growth of F. oxysporum, compared with the F. oxysporum colony area in the absence of *Hanseniaspora*, was reduced to 55% in SC medium lacking pantothenate (-PA) and to 33% in complete SC medium.

In these assays, *H. meyeri* (APC 12.1) grew only around the F. oxysporum colony and, to some degree, in the vicinity of the tomato root (Fig. 1A). In contrast, this yeast grew on the entire plate surface and inhibited Fusarium in competition assays on peptone-dextrose agar (PDA) and complete yeast nitrogen base (YNB) medium (see Fig. S1 in the supplemental material). This result suggests that the yeast must obtain an essential nutrient from the fungal colony or the plant root when plated on Murashige and Skoog (MS) medium that was used for the *in vitro* cultivation of plants. Independent *Hanseniaspora* growth tests were performed with the same medium and with radish seedlings or with the filamentous fungi Penicillium polonicum, Mucor moelleri, or Botrytis caroliniana. In all assays, the same yeast growth pattern was observed, as follows: *H. meyeri* colonies were growing in the vicinity of a competing fungus or a host plant, and no colonies were observed further away from either organism (see Fig. S2 in the supplemental material). These observations suggested that, on MS medium, *H. meyeri* obtains an essential nutrient from competing fungi or from plants.

### *H. meyeri* (APC 12.1) is auxotrophic for pantothenate.

In order to identify the essential nutrient(s) that *H. meyeri* obtains from fungi or plants, growth experiments with different defined media were performed. While the yeast did not grow on MS medium, it grew well on a synthetic complete (SC) medium containing yeast nitrogen base (YNB) with a complete supplement mix. MS medium lacks several vitamins that are present in standard microbiological culture media. To identify which vitamin the *H. meyeri* strain APC 12.1 obtains from fungi or plants, we performed growth assays with amino benzoic acid (ABA), biotin, calcium pantothenate, folic acid, and riboflavin.

*H. meyeri* grew well in the absence of either ABA, folic acid, or riboflavin and showed reduced growth in medium lacking biotin but was unable to grow in the absence of calcium pantothenate (Fig. 1B). Similarly, *H. meyeri* growth on MS medium could be recovered by adding calcium pantothenate, although colonies were relatively small. On SC medium lacking pantothenate, *H. meyeri* grew only in the vicinity of the F. oxysporum colony, as was observed on MS medium (Fig. 1C). The *Hanseniaspora* cells growing around F. oxysporum inhibited Fusarium growth to 55% (compared with growth in the absence of any yeast), while on complete SC medium, the Fusarium colony was reduced to 33% of the control (Fig. 1D). These findings suggested that *H. meyeri* (APC 12.1) is unable to synthesize pantothenate and must obtain this vitamin from the environment.

### Biotin and calcium pantothenate are required for biocontrol activity of *Hanseniaspora* against Fusarium.

**FIG 2:**
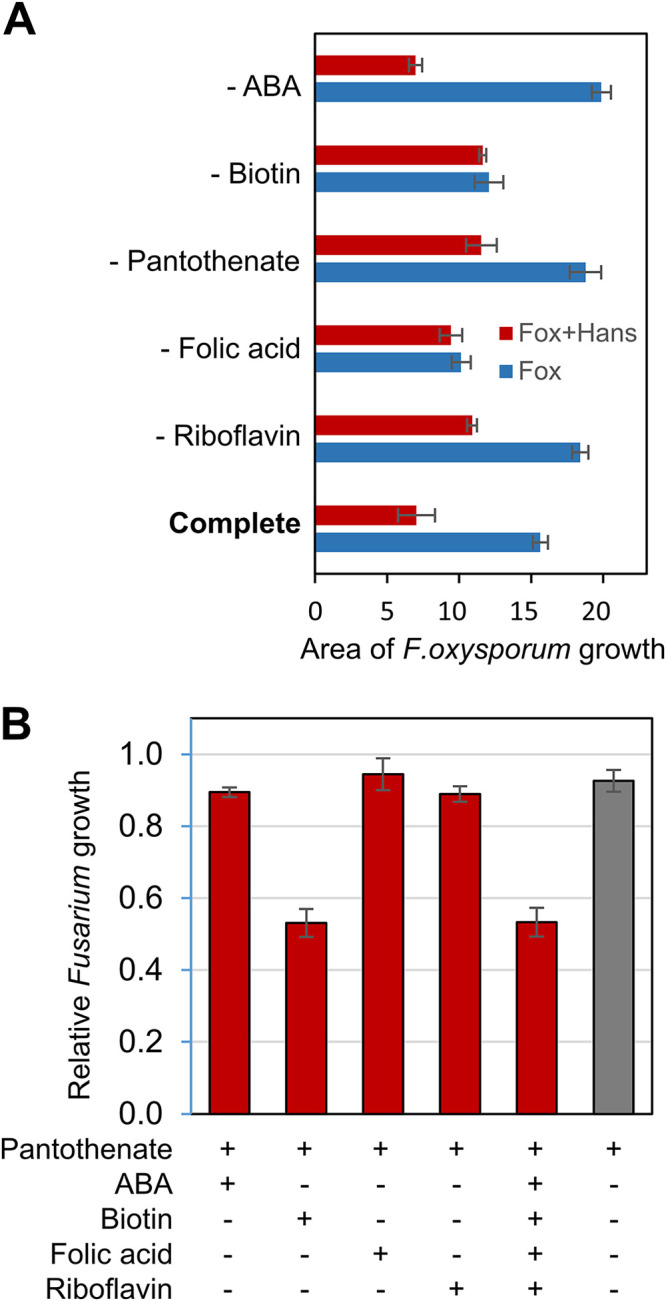
Biotin and calcium pantothenate are required for biocontrol activity of *Hanseniaspora* against Fusarium. (A) Absence of vitamins in SC medium affects antagonistic activity. In complete SC medium or when amino benzoic acid (ABA) is lacking, *Hanseniaspora* strongly inhibits the growth of Fusarium (red) compared to the control without the yeast (blue). The activity is reduced without calcium pantothenate or riboflavin and abolished in the absence of either biotin or folic acid. (B) Addition of biotin and calcium pantothenate to MS medium results in the same inhibitory activity as in the medium with the five vitamins. Supplementation with pantothenate and either one of the other vitamins did not improve inhibitory activity.

To test the effect of vitamins on the biocontrol activity of *H. meyeri* against Fusarium, competition assays on different MS and SC media were performed. F. oxysporum growth was not inhibited by the presence of *H. meyeri* on SC media that specifically lacked either biotin or folic acid, suggesting that *Hanseniaspora* growth alone (in SC medium lacking folic acid) does not always result in inhibition of Fusarium (Fig. 2A). However, *H. meyeri* inhibited F. oxysporum growth in the absence of ABA, calcium pantothenate, or riboflavin (compared with growth in the absence of the yeast). In the presence of *H. meyeri*, F. oxysporum growth was reduced by more than 50% on complete SC medium and in the absence of ABA, while this inhibition was lower when either calcium pantothenate or riboflavin were omitted (Fig. 2A). On MS medium, the addition of calcium pantothenate alone did not restore the antagonistic activity of *H. meyeri* (Fig. 2B). We therefore supplemented MS medium with pantothenate and each of the other four vitamins that were tested before and performed competition assays. Only the addition of pantothenate and biotin resulted in strong F. oxysporum inhibition by *H. meyeri* (over 40%) (Fig. 2B). These two vitamins were sufficient to recover inhibitory activity to the same degree as the addition of all vitamins, most likely because the lack of biotin and pantothenate affected *H. meyeri* growth the most (Fig. 1B).

These results demonstrate that biotin and calcium pantothenate are required for *H. meyeri* biocontrol activity against F. oxysporum. Even though *H. meyeri* is auxotrophic for pantothenate, it inhibited F. oxysporum on SC medium lacking only calcium pantothenate. This yeast must thus possess effective transporters that can take up vitamin secreted from F. oxysporum mycelium.

### The *H. meyeri* (APC 12.1) genome lacks crucial genes associated with pantothenate biosynthesis.

**FIG 3:**
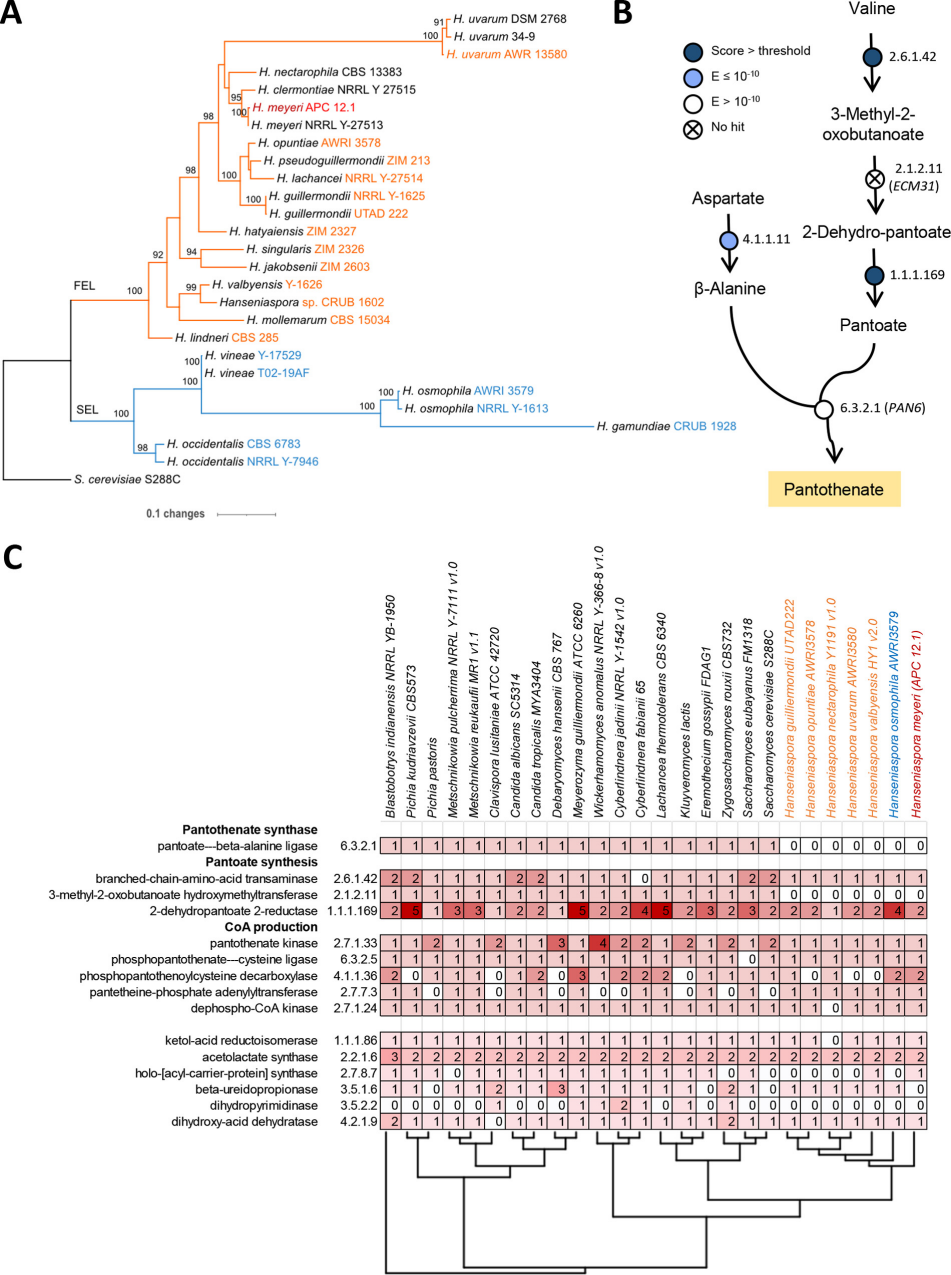
The *Hanseniaspora meyeri* (APC 12.1) genome lacks crucial genes associated with pantothenate biosynthesis. (A) Core genome CDS single nucleotide polymorphism (SNP)-based phylogeny of 26 *Hanseniospora* strains, including S. cerevisiae S288C as an outgroup. The general topology of the phylogeny described in Steenwyk et al. (13) was conserved and the *H. meyeri* APC 12.1 strain described in this study clustered with the previously sequenced *H. meyeri* NRRL Y-27513 strain. The *x* axis and the scale bar display substitutions per site on the core genome CDS SNP alignment. The numbers of the branches indicate bootstrap values of 100 bootstrap replicates and values of <90 are not shown. The accession numbers of the 27 yeasts genomes are listed in Table S5 in the supplemental material. (B) The pantothenate biosynthesis pathway from S. cerevisiae showing enzymes with a homolog in *H. meyeri.* The significance of the best BLAST hit is indicated in white (E > 10-10 or low significance), light blue (E ≤ 10-10), or dark blue (highly significant). (C) Presence of genes in the pantothenate biosynthesis pathway in a selection of budding yeasts (Saccharomycotina). The number of genes per enzyme category is highlighted in shades of red (0 is white and 5 the darkest red). Selected representatives of the subphylum and six *Hanseniaspora* species were included along with the isolate APC 12.2. *Hanseniaspora* species from the fast-evolving lineage are shown in orange, while slow-evolving strains are depicted in blue (as described by Steenwyk et al. [13]). The tree corresponds to the phylogenetic relationships of the selected species. The mentioned absence of two key genes for pantothenate biosynthesis occurs in all *Hanseniaspora* representatives. Meanwhile, homologous genes for the steps upstream of pantothenate are found in all other yeasts except Cyberlindnera fabianii, which lacks the branched-chain-amino-acid transaminase (EC 2.6.1.42). Downstream from pantothenate, the coenzyme A (CoA) production pathway shows the absence of a variety of genes across the tree.

In order to identify the genetic basis for pantothenate auxotrophy and the ability to grow with externally supplied pantothenate, the genome of *H. meyeri* (APC 12.1) was sequenced using a combination of long (Pacific Biosciences and Oxford Nanopore Technologies) and short (Illumina) reads, *de novo* assembled, and analyzed. The final genome assembly consisted of seven complete chromosomes and a 17-kb mitogenome (see Table S1 in the supplemental material). The mitogenome showed inverted repeats, which suggests a linear mitochondrial genome, as it has been observed in other yeasts (15–17). In our genome assembly, on chromosome 2 (approximately at position 1060 kb), two 96% identical tandem rDNA units were present. Based on the YeastIP database, the first rDNA unit was 96% (internal transcribed spacer [ITS]) and 95% (D1/D2 region) identical to Hanseniaspora clermontiae and *H. meyeri*, respectively, while the second unit showed 99% identity to the corresponding sequences from both species. The large *MDN1* gene (open reading frame [ORF] HANS 0B04920, at 950 kb on chromosome 2) was 99% identical to the corresponding gene from *H. meyeri* but only 93% identical to *H. clermontiae*. A core genome analysis of our isolate APC 12.1 genome and 25 other, previously published *Hanseniaspora* genomes, was created and a maximum likelihood phylogenetic tree was generated (Fig. 3A). Based on this analysis, the isolate APC 12.1 clustered together with *H. meyeri* Y-27513. The isolate APC 12.1 is thus named and referred to as *H. meyeri*.

The pantothenate biosynthetic pathway starts with l-aspartate and l-valine as the substrates and results in the formation of pantoate and β-alanine, which are the substrates for the final reaction catalyzed by pantothenate synthase (Fig. 3B). The *H. meyeri* APC 12.1 genome contained homologs of most genes involved in this pathway, but we could not detect a 3-methyl-2-oxobutanoate hydroxymethyltransferase gene (EC 2.1.2.11, *ECM31* in S. cerevisiae) and only an insignificant hit (E = 0.41, score = 34) for pantothenate synthase (EC 6.3.2.1, *PAN6* in S. cerevisiae). We thus concluded that *H. meyeri* APC 12.1 also lacks the *PAN6* gene. A genome analysis of different *Saccharomycetales* species showed the absence of *PAN6* (EC 6.3.2.1) and *ECM31* (EC 2.1.2.11) homologs in all *Hanseniaspora* genomes available at Mycocosm, while all other selected yeasts (model organisms, human pathogens, and yeasts with biotechnological or biocontrol potential) harbored genes corresponding to all the enzyme types upstream of pantothenate biosynthesis in S. cerevisiae (Fig. 3C). The absence of these two genes makes it impossible for *H. meyeri*, and other isolates of this genus, to synthesize pantothenate and is thus the molecular cause for the observed pantothenate auxotrophic phenotype.

### The *H. meyeri* (APC 12.1) genome harbors six putative pantothenate transporter genes.

Pantothenate is a crucial precursor of CoA and thus is essential for various metabolic functions, including the biosynthesis of fatty acids and sterols in the citric acid cycle and gene regulation through histone acetylation. A cell that is unable to synthesize pantothenate thus must possess transporters that allow uptake of this vitamin. In order to identify potential pantothenate transporters in *H. meyeri*, we performed BLAST searches with the sequence of the S. cerevisiae pantothenate transporter Fen2 and searched KEGG annotations.

**FIG 4:**
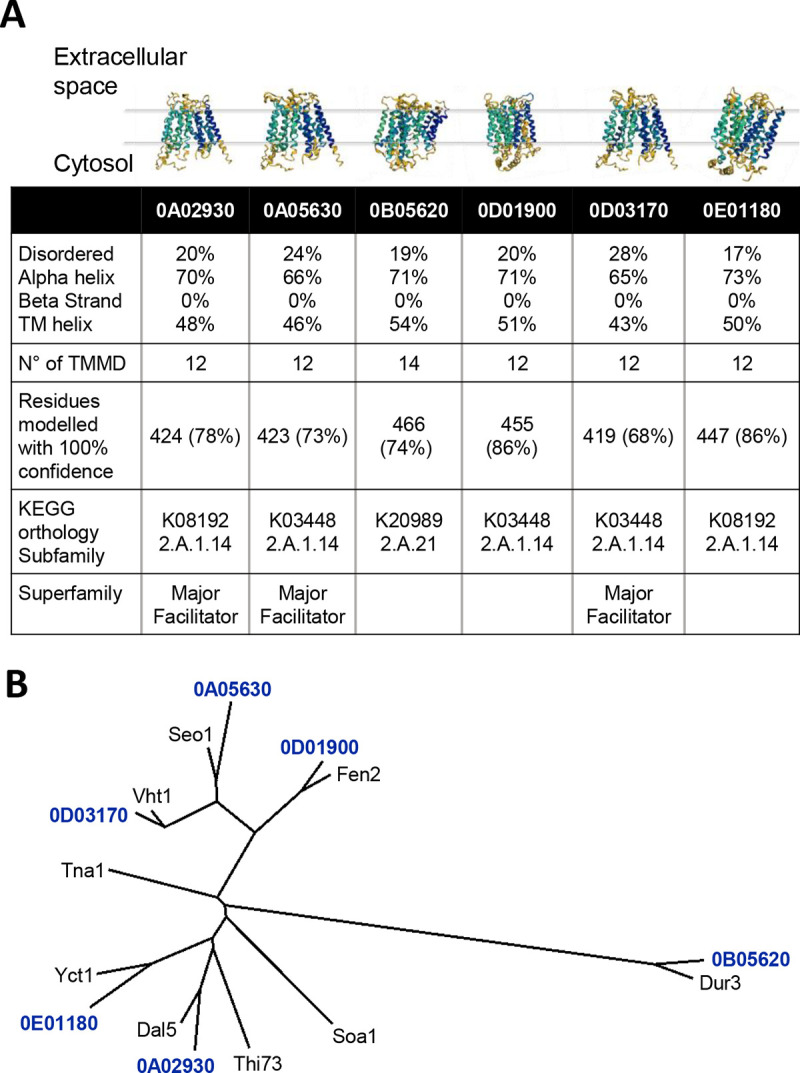
The *Hanseniaspora meyeri* (APC 12.1) genome harbors six putative pantothenate transporter genes. (A) Amino acid sequences of the six transporters were analyzed with Phyre2. All six predicted proteins are formed by 65% to 73% alpha helixes, of which many (43% to 54% of the total protein) form the transmembrane domains (TMMD). The number of transmembrane domains is predicted to be 12 for all the transporters except for 0B05620, for which 14 such domains are proposed. (B) Sequence similarity of the six putative *Hanseniaspora* pantothenate transporters with their corresponding best BLAST hits in the S. cerevisiae genome.

Based on BLAST searches, four hits for the Fen2 sequences were obtained (the ORFs 0D01900, 0D03170, 0A05630, and 0A02930). The KofamKOALA KEGG analysis of the annotated *H. meyeri* APC 12.1 genome predicted potential pantothenate transporter function for the same four ORFs and for two additional genes (ORF numbers 0B05620, and 0E01180). In total, we have thus identified six genes with potential pantothenate transporter function (Fig. 4A). These genes encoded proteins ranging in size from 419 to 466 amino acids. Structural predictions, based on the amino acid sequences, estimated all of the six proteins to be α-helical bundles. Except for the protein encoded by 0B05620 (14 transmembrane domains, N and C terminus facing the extracellular space), 12 transmembrane domains and cytosolic N and C termini were predicted for all proteins. The *H. meyeri* ORFs 0A02930, 0A05630, and 0D03170 were assigned to the major facilitator superfamily and the glycerol-3-phosphate transporter family. The sole S. cerevisiae pantothenate transporter Fen2 also belongs to the major facilitator superfamily and, as most members of this superfamily, has 12 transmembrane helixes. However, it belongs to the allantoate permease family, a group of yeast transporters importing anionic vitamins (18, 19). Reciprocal BLAST searches of the *H. meyeri* sequences for 0A02930, 0A05630, 0B05620, 0D01900, 0D03170, and 0E01180 against the S. cerevisiae genome revealed the transporters Dal5, Seo1, Dur3, Fen2, Vht1, and Yct1, respectively, as the best hits (Fig. 4B; see Table S2 in the supplemental material). The transporters Soa1 and Tna1 were also significant blast hits (for 0A02930, 0D01900, and 0D03170).

Based on genome analyses, we thus identified six *H. meyeri* (APC 12.1) genes as candidates for encoding a functional pantothenate transporter that enable this yeast to grow and compete in the environment.

### Complementation with *H. meyeri* 0D01900 restores pantothenate uptake in S. cerevisiae.

S. cerevisiae possesses a single pantothenate transporter, Fen2, and is also able to synthesize the vitamin. However, if pantothenate is absent from glucose-containing media, growth is slightly impaired, suggesting that pantothenate uptake is limiting under these conditions. A Δ*pan6* deletion mutant that lacks the enzyme (EC 6.3.2.1) required for the final step of pantothenate synthesis (Fig. 3A) has a comparable phenotype as *H. meyeri* APC 12.1 (i.e., it does not grow without pantothenate but can obtain the vitamin from other fungi). In a Δ*pan6* strain background, the Fen2 transporter is essential because pantothenate cannot be synthesized and must therefore be taken up. This strain can thus be taken advantage of to create a reporter strain for pantothenate transport. For this purpose, the *FEN2* promoter was exchanged with the galactose inducible promoter GALL, and putative *Hanseniaspora* pantothenate transporter genes were heterologously expressed by integrating the corresponding genes at the *URA3* locus (see Fig. S3 in the supplemental material).

**FIG 5:**
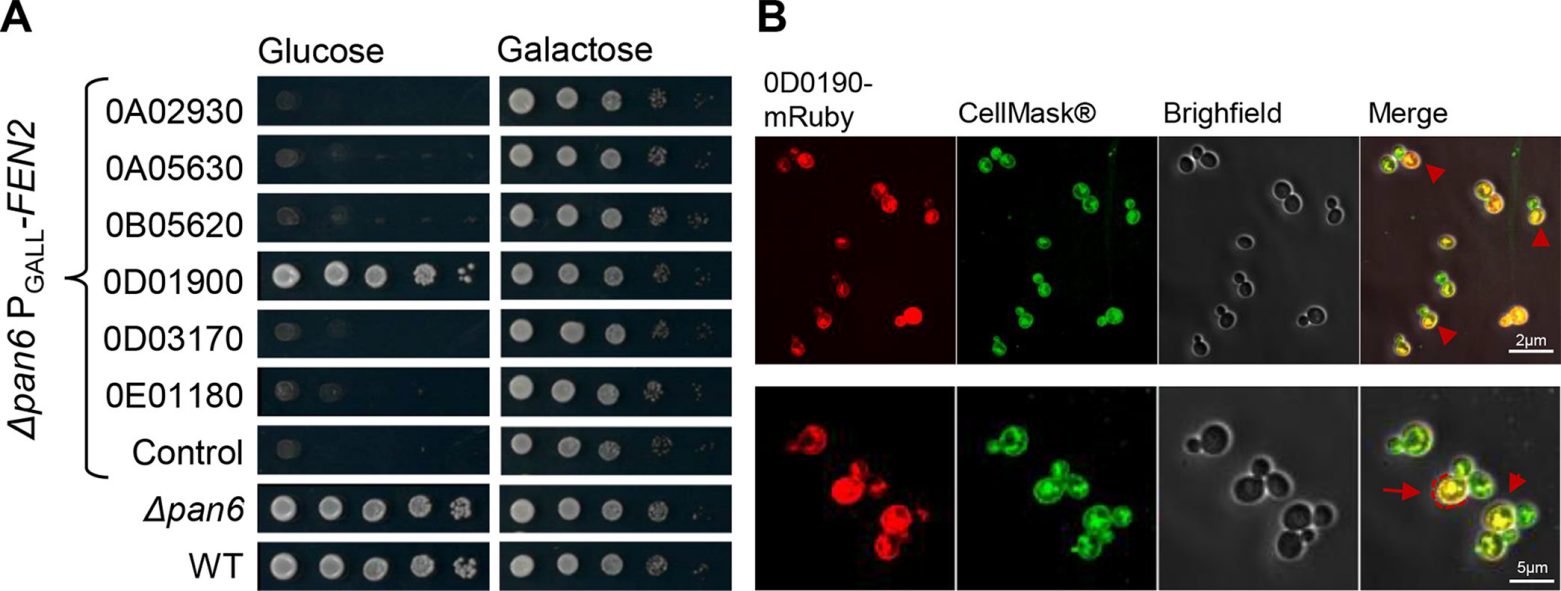
Complementation with the *H. meyeri* gene 0D01900 restores pantothenate uptake in S. cerevisiae. Heterologous expression of 0D01900 restores growth in the S. cerevisiae reporter strain. (A) Six predicted *H. meyeri* pantothenate transporters were constitutively expressed in the Δ*pan6* P_GALL_*-FEN2* reporter strain, which is unable to both produce pantothenate and obtain it from the medium when glucose is the carbon source. Out of the six transporters, only 0D01900 expression is able to recover normal growth in glucose. (B) 0D01900 localizes to the plasma membrane of mother cells, as confirmed by its colocalization with the CellMask green plasma membrane dye.

As expected, the reporter strain grew in a comparable manner as wild-type yeast and showed no growth defect in YNB medium containing galactose (“control” in Fig. 5A), while it was unable to grow with glucose as the sole carbon source. Constructs of the six potential *H. meyeri* (APC 12.1) pantothenate transporter genes (untagged, 3xHis + 6xFLAG-, or mRuby-tagged, codon optimized for S. cerevisiae) were transferred and expressed under the constitutive P_TDH3_ promoter in this reporter strain. Western blot analysis confirmed the expression of three of the six transporters encoded by the genes 0A05630, 0D01900, and 0D03170 (see Fig. S4 in the supplemental material). Spot assays showed that only 0D01900 was able to recover growth of the reporter strain on glucose-containing medium, suggesting that the corresponding protein is a pantothenate transporter with a homologous function to S. cerevisiae Fen2. We thus name the gene 0D01900 from *H. meyeri* APC 12.1 *FEN2*. This result was the same for the cells harboring the tagged and untagged constructs. Confocal microscopy localized the mRuby-Fen2 fusion protein to the plasma membrane, as confirmed by colocalization with a green fluorescent plasma membrane dye (Fig. 5B). Overall, these results show that the Fen2 protein encoded by the gene *OD01900* from the auxotrophic isolate *H. meyeri* APC 12.1 functions as a pantothenate transporter in S. cerevisiae. However, we cannot exclude pantothenate transporter function in *Hanseniaspora* for the other five proteins because the genes may not have been expressed or the proteins may not have been stable, correctly localized, or functional in S. cerevisiae.

## DISCUSSION

The *H. meyeri* strain APC 12.1 was identified originally as one of the most strongly antagonistic yeasts in a screen of 40 different species against 16 filamentous fungi (14). The study presented here describes the unexpected discovery that this strain is a naturally occurring pantothenate auxotroph. Auxotrophic microorganisms might seem an unlikely choice for applications such as biocontrol because of their metabolic limitations. However, the use of such strains may constitute a natural biocontainment strategy and mitigate concerns about uncontrolled propagation in the environment. Natural auxotrophs should thus be easier to register as biocontrol agents, while their metabolic limitations can likely be overcome by strain-specific formulations. Based on the observations provided here, specific formulations and application strategies that include calcium pantothenate, but also other vitamins, such as biotin and folic acid, could be envisioned. On the other hand, it could also be argued that the observation of a naturally occurring auxotrophic yeast proofs that this property does not prevent spread in the environment and thus potentially negative side effects resulting from the release in the environment. Interestingly, biocontainment strategies are actively studied and developed in order to prevent the spread of synthetic organisms (or “normal” genetically modified microorganisms) in the environment (20–22). Such tools are considered essential for possible applications of synthetic organisms and are thus of considerable economic interest (23, 24).

The *H. meyeri* (APC 12.1) genome lacks key genes for the biosynthesis of pantothenate, which is likely the root cause of the observed pantothenate auxotrophy. Such a pantothenate auxotrophy has also been described in the bacterial species Zymomonas mobilis and *Methylobacterium* spp. (25, 26). In these examples, auxotrophy was due to the lack of the corresponding aspartate decarboxylase PanD or one of the enzymes required to synthesize the precursor β-alanine. Interestingly, these bacteria are also found associated with plants and in fermentation processes, suggesting that these environments are able to sustain the growth of pantothenate auxotrophs. Similarly, pantothenate auxotrophy (and also auxotrophy for biotin, niacin, and thiamine) is frequent among bacteria living in the Arabidopsis thaliana phyllosphere (27). However, in yeasts, pantothenate auxotrophy has been described only for S. cerevisiae strains isolated from sake and is due to a loss of function in the Ecm31 gene, which impedes the synthesis of pantoate (28). A recent study isolated 433 wild yeasts from a variety of raw and fermented cereals. All isolates, belonging to 9 species, grew in the absence of pantothenate. In contrast, biotin and riboflavin were necessary for the growth of 64% and 14.3% of all strains, respectively (29). Pantothenate auxotrophy is thus rare among microorganisms and has not been described for environmental fungi. Although only a few reports are available, the secretion of pantothenate or pantothenate precursors seems a common phenomenon, as it has been described for Arabidopsis thaliana, root exudates from different plants, Cryptococcus neoformans (as a quorum-sensing signal), or even infected lung epithelial cells (26, 30–32). Pantothenate therefore seems to be readily available in plant environments, which likely explains why this auxotrophy can arise in plant-associated yeasts and bacteria.

The genus *Hanseniaspora* is characterized by some of the smallest genome sizes and lowest gene numbers in budding yeasts. A genomic study with 25 genomes from 18 *Hanseniaspora* species described a faster- and a slower-evolving *Hanseniaspora* lineage and uncovered large-scale gene loss as a means of genome evolution in both (13). With these gene losses, a number of metabolic functions, as well as cell cycle checkpoints, disappeared. Gene functions related to growth in and fermentation of maltose, degradation of sucrose, and assimilation of galactose are often absent. Several *Hanseniaspora* species are thus not able to grow with maltose, raffinose, melezitose, galactose, or sucrose as the sole carbon sources. Another vitamin, thiamine, is also not synthesized by some species, as most of the biosynthetic pathway has been lost. The species *H. meyeri* belongs to the faster-evolving *Hanseniaspora* lineage and is shown here to be metabolically restricted and dependent on cross-feeding of essential nutrients from plants and competing microorganisms. Although an auxotrophic phenotype may seem a competitive disadvantage, the loss of biosynthetic genes can confer a selective advantage to bacteria and stabilize microbial communities (33–37). In addition, the absence of components of the cell cycle and the reduced genome size are thought to accelerate cell division and growth, explaining the high cell densities reached rapidly in fermentation products. The combination of these two mechanisms could thus explain the strong biocontrol activity and ability to compete in the environment that we describe here for the *H. meyeri* APC 12.1 strain. Experiments with synthetic communities and *Hanseniaspora* strains that were complemented with a functional pantothenate biosynthesis pathway (which is not possible at the moment due to our inability to transform *H. meyeri* APC 12.1) would be required to confirm if this is an example for the “competition for nutrients” mode of action that is, counterintuitively, based on and made possible by vitamin auxotrophy.

In general, competition for nutrients and space has been suggested as the main mechanism of yeast biocontrol against fungal pathogens (9). Yeasts grow faster and colonize the available space, take up and deplete essential nutrients, and reduce pathogen growth (37). However, few studies describe this phenomenon in detail or at a molecular level (9). Here, we describe auxotrophy for the vitamin pantothenate. Auxotrophies, such as the one described here, and the high competitiveness in general, could also be taken advantage of for biocontrol applications in agriculture. Including nutrients and vitamins in the formulation of biocontrol yeasts could improve the establishment and persistence of these organisms on the crop and thus result in a more reliable biocontrol activity. Detailed analyses of the metabolic capabilities and uptake efficiencies for different nutrients may thus greatly benefit biocontrol and lead to new and more successful applications of antagonistic yeasts in crop protection.

## MATERIALS AND METHODS

### Strains and cultivation.

All strains used in this study are listed in Table S3 in the supplemental material. *H. meyeri* (APC 12.1) was isolated from apple flowers in Switzerland (14). For competition assays, Fusarium oxysporum f. sp. *lycopersici*, kindly provided by Antonio Di Pietro, was used. Tomato seeds (cultivar Moneymaker) were surface sterilized and germinated on Murashige and Skoog (MS) agar. The S. cerevisiae Δ*pan6* deletion strain was obtained from EUROSCARF.

Interaction assays with *H. meyeri*, F. oxysporum, and tomato seedlings were performed on MS agar (Duchefa Biochemie, Haarlem, Netherlands). Overnight *H. meyeri* cultures were collected, the pellet was washed with water, and 100 μL of yeast solution (optical density at 600 nm [OD_600_], ≈0.1) was spread onto the plate. F. oxysporum conidia were isolated after growth for 3 to 4 days in potato dextrose broth (PDB; Becton, Dickinson and Company, Le Pont de Claix, France) by filtering through two layers of Miracloth (Merck Millipore, Schaffhausen, Switzerland). Conidia were collected and washed twice with water, the concentration was estimated by hemocytometer counting, and solutions with 5 × 10^5^ conidia/mL were prepared. A 5-μL drop of conidia solution was placed on one side of the plate and 1-week-old plants were placed on the other side. Competition assays with *H. meyeri* and F. oxysporum in the presence or absence of different vitamins were performed with SC medium (Formedium, Norfolk, UK) and as described previously (14).

### Genome sequencing and analysis.

*H. meyeri* (APC 12.1) genomic DNA was extracted using a phenol-chloroform extraction protocol and as stated previously (38, 39). Cells from an overnight culture were collected and resuspended in 200 μL of Harju buffer (2% Triton X-100, 1% SDS, 100 mM NaCl, 10 mM Tris-HCl [pH 8.0], and 1 mM EDTA). Disruption was performed by two rounds of freezing with liquid nitrogen and boiling at 95°C. A high-coverage genome was produced using a combination of Oxford Nanopore Technologies (ONT), Illumina, and PacBio technologies. Libraries were prepared using the Nanopore ligation sequencing kit and the Illumina Nextera XT DNA library prep kit. After filtering the PacBio and Oxford Nanopore subreads with Fitlong (v0.2.0) and the Illumina reads with Trimmomatic (v0.39), we performed a *de novo* assembly of PacBio reads with Flye (v2.4). Contigs were then polished using the Illumina and ONT reads. For the mitogenome, a reference-based approach was followed. The mitogenome sequence of *Hanseniaspora uvarum* (DQ058142) was downloaded from NCBI, and PacBio reads were mapped to the reference using minimap2 (set parameters: -a, -x map-pb). Mapping reads were filtered from the bam file using SAMtools (-F 4) and were extracted into a fastq file using bam2fastq (v1.1.0). The reads were assembled using Flye (v2.4; default parameters, except: estimated genome size of 20 kb [40]), which resulted in the assembly of the 17-kb linear mitogenome. PlasmidSpades (41), run on the Illumina data, did not detect plasmids. The mean telomere length (pattern “CCTGA”) was calculated using the Illumina reads and Computel (v1.2) (42) and was estimated to be 2,275 bp. The number of telomere patterns at both ends of each contig was counted manually (Table S1). ploidyNGS (v3.1.2) (43) as well as nQuire (44) estimated the genome to be diploid. The extensive polishing and manual curation resulted in a total of 7 chromosomes and 1 mitogenome. The total genome size was 8,767,711 bp.

*H. meyeri* (APC 12.1) genes were identified as described previously by using the Yeast Genome Annotation Pipeline (YGAP) (38). KOALA was used to assign KEGG Orthologs (KOs; K numbers) to the predicted proteins (45). The KEGG Mapper Reconstruct tool was used to assign the KOs to pathway modules (46).

A core genome alignment and a maximum likelihood phylogenetic tree were created on coding DNA sequences (CDSs) using PHaME as described by the developers (47).

### Cloning and transformation.

Potential pantothenate transporter genes were synthesized *in silico* (Twist Bioscience, South San Francisco, USA; codon optimized for S. cerevisiae and lacking the restriction sites Bsa I, BsmB I, and Not I, verified by sequencing) and were introduced into expression vectors using Golden Gate cloning (48) (MoClo Yeast Toolkit; Addgene, Watertown, MA). Each transporter gene was cloned with the pTDH3 promoter and tTDH1 terminator modules into a green fluorescent protein (GFP) drop-out vector containing the *HIS3* gene for selection of transformants 3′- and 5′-*URA3* homology regions for genomic integration (Fig. S3). The reaction was performed under temperature cycles of 37°C for 2 min and 16°C for 5 min using the restriction enzyme Bsa I-HF V2 and the Hi-T4 DNA ligase (from New England BioLabs; Bioconcept, Allschwil, Switzerland), followed by a final 10-min ligation at 16°C. Intermediate and final constructs were transformed into Escherichia coli DH5α and were purified with the Qiaprep spin miniprep kit (Qiagen, Hombrechtikon, Switzerland). The size of the final products was confirmed by restriction digestion with NotI and gel electrophoresis. The identity of each gene within the final constructs was confirmed by sequencing with a primer in the pTDH3 region. The resulting alignments spanned ~450 bp of the promoter and ~650 of each pantothenate transporter gene.

The *FEN2* promoter of Δ*pan6* S. cerevisiae mutants was exchanged by transformation with a PCR product containing the inducible pGALL promoter as described by Janke et al. (49). Briefly, S1 and S4 primers were designed with homology to the *FEN2* promoter region for amplification of the GALL promoter and by using the plasmid pYM-N27 from the PCR toolbox (Euroscarf/Scientific Research & Development GmbH, Oberursel, Germany) as a template (49). Cells were selected in YPD with nourseothricin (100 μg/mL; Jena Bioscience, Jena, Germany) after incubation at 30°C for 3 days. All primers used in this study are listed in Table S4 in the supplemental material.

Golden Gate constructs (48) containing the putative pantothenate transporter genes were linearized with NotI for integration into the *URA3* locus of the S. cerevisiae genome. After lithium acetate transformation, cells were grown in yeast nitrogen base without histidine and with galactose as the carbon source.

### Fluorescence microscopy.

Cells were grown overnight at 30°C in YPD medium, diluted to an OD_600_ of 0.1, and harvested when the culture reached 0.5. Cells were spread onto slides coated with an SC agar patch. Stacked images were recorded at a spinning disc confocal inverted microscope (Zeiss Axiovert 200m) using the 100× oil objective and an Evolve EMCCD camera (Photometrics).

### Western blot.

S. cerevisiae strains expressing constructs tagged with 3×FLAG-6×His were grown overnight in liquid synthetic complete medium with 2% galactose (Formedium, Norfolk, UK), diluted to an OD of 0.1, and cultured again to exponential phase (OD of 0.5). For protein extraction, approximately 10^8^ cells were washed with sterile water and suspended in 200 μL lysis buffer containing 0.1 M NaOH, 0.05 M EDTA, 2% SDS, and 2% β-mercaptoethanol. Cells were then boiled at 90°C for 10 min, 5 μL of 4 M acetic acid was added, and the sample was boiled again for 10 min. Concentration was measured with the Qubit protein assay following the manufacturer instructions, and ~50 μg of the protein was loaded to 8% bis-tris mini protein gels (Invitrogen Bolt) with a 0.25 M Tris-HCl (pH 6.8), 50% glycerol, and 0.05% bromophenol blue loading buffer. Electrophoresis using bis-tris gels was performed according to manufacturer’s instructions with morpholinepropanesulfonic acid (MOPS) buffer, and proteins were transferred to a nitrocellulose membrane. The 6×-His tag monoclonal antibody (HIS.H8; Invitrogen) and goat anti-mouse IgG (H+L) horseradish peroxidase (HRP) (Invitrogen) were used to probe the membrane as primary and secondary antibodies, respectively. Detection was done using the Novex ECL chemiluminescent substrate reagent kit (Invitrogen).

### Data availability.

The genome annotation and all sequencing data are available at the Harvard Dataverse (https://dataverse.harvard.edu/dataverse/Hans_genome) and NCBI under the BioProject PRJNA907227.
