# Supplementary material for: Pantothenate Auxotrophy in a Naturally Occurring Biocontrol Yeast
Source: Appl Environ Microbiol. 2023 Jul 5;89(7):e00884-23. doi: 10.1128/aem.00884-23 (PMC10370309; doi:10.1128/aem.00884-23)
Supplement: Supplemental file 1 — Supplemental material. Download aem.00884-23-s0001.pdf, PDF file, 0.5 MB [file aem.00884-23-s0001.pdf]

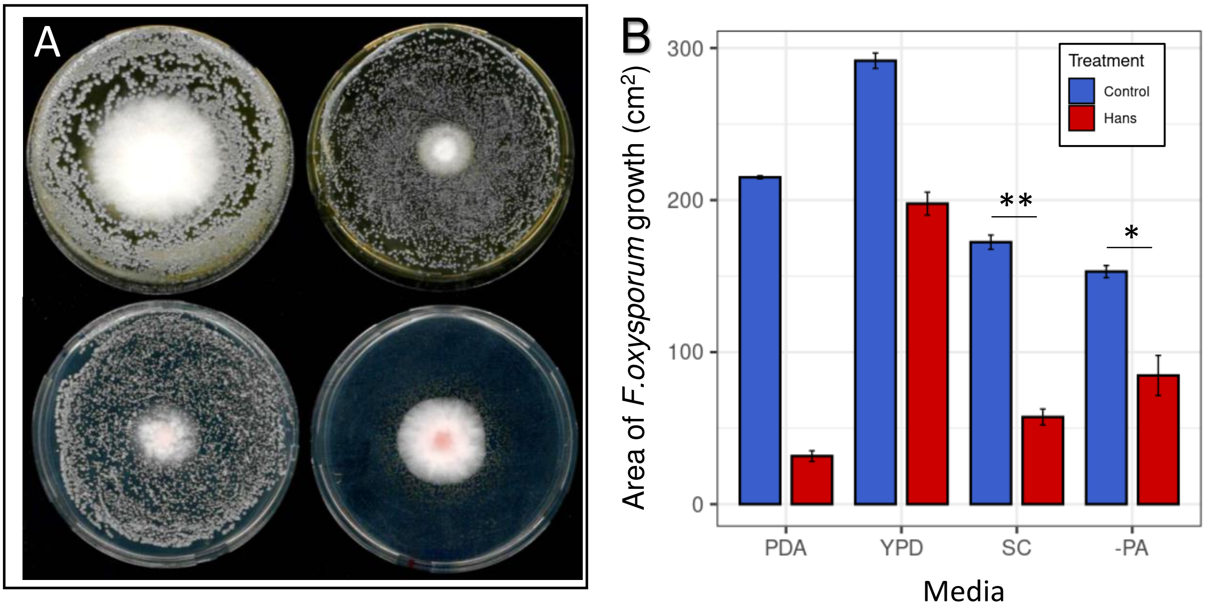

Supplementary Figure 1

A. Competition between *Hanseniaspora meyeri* and *F. oxysporum* f. sp. *lycopersici* in YPD, PDA, YNB and YNB without pantothenate media (from top left to bottom right). *H. meyeri* grew on the entire surface in all media, except when pantothenate was lacking. The *F.oxysporum* colony was larger in YPD, but growth was reduced in all media. **B**. Control of *F.oxyporum* was the strongest in PDA; the growth area was reduced to 15% as compared to the plate without *H. meyeri*. In SC media with and without pantothenate, the *Fusarium* growth was reduced to 33% and 55% (p.adj FDR method; -PA *: 0.027, SC **: 0.00018.

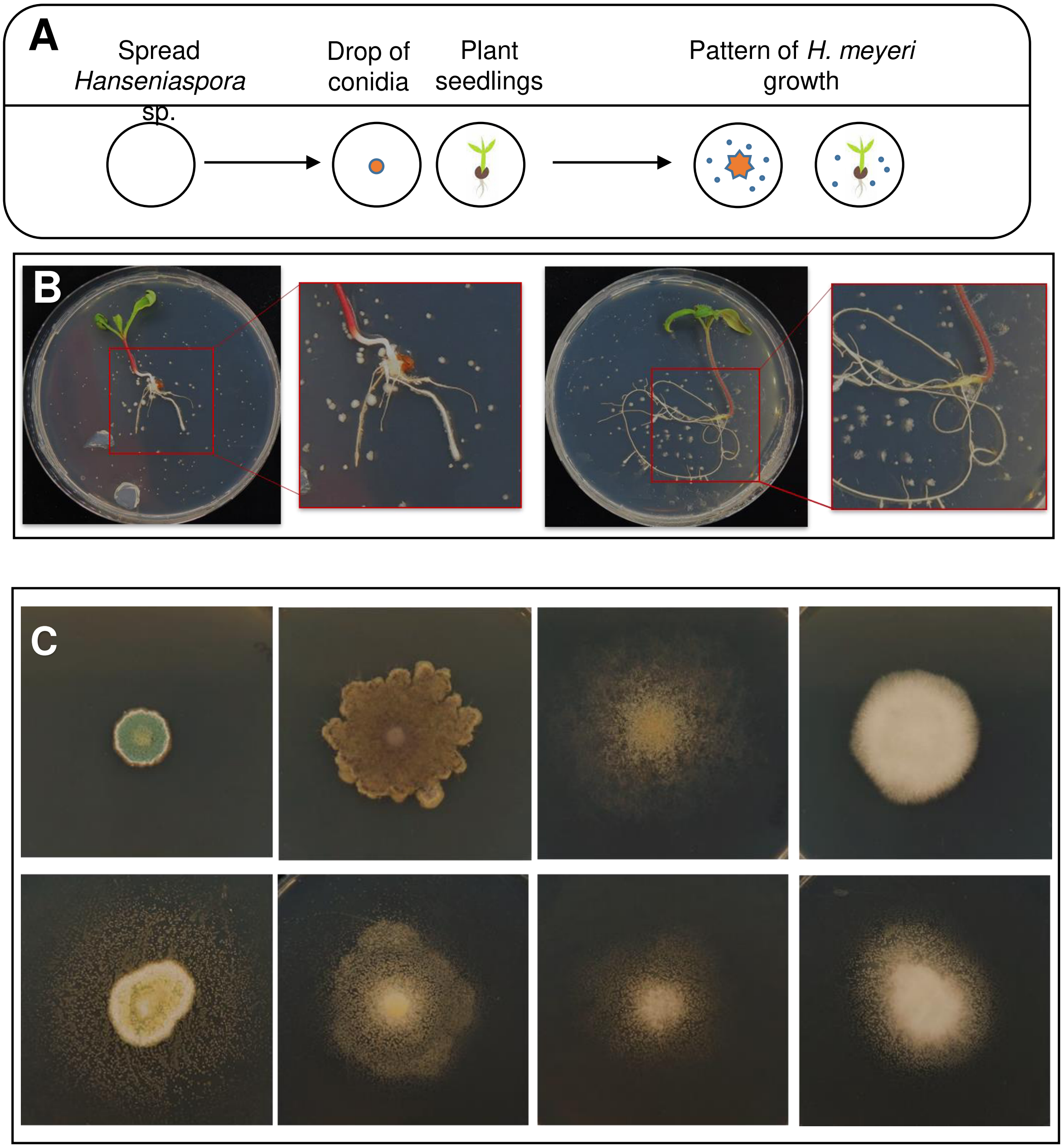

Supplementary Figure 2

A. Diagram of co-culture assays between *H. meyeri* APC 12.1 and filamentous fungi or plant seedlings in MS medium **B.** Co-culture with radish and tomato plants and **C.** competiton assays with four plant pathogenic fungi in MS medium show that *H. meyeri* only grew in the vicinity of other the competing fungi. **Top row** : Filamentous fungi colony growing alone, **Bottom row** : *H. meyeri* APC 12.1 and fungi competition assay. From right to left: *Penicillium polonicum, Mucor moelleri, Botrytis caroliniana, Fusarium oxysporum.*

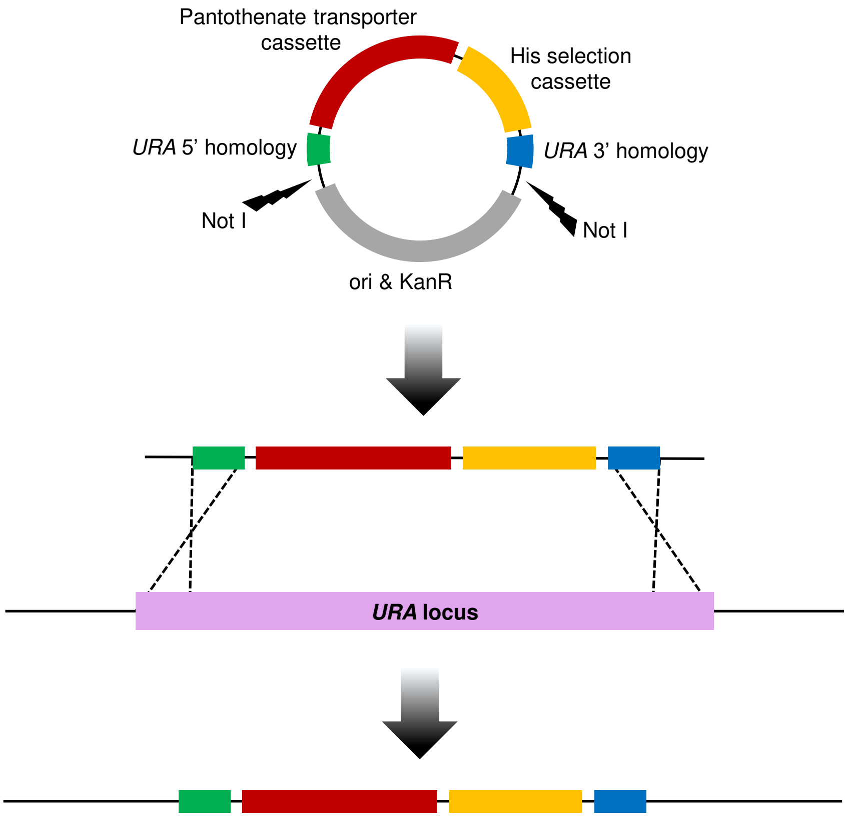

Supplementary Figure 3. Potential *H. meyeri* pantothenate transporters were cloned (with the P_{TDH3} promoter and $T_{TDH}1$ terminator) into a vector containing a *HIS3* marker gene, 3'- and 5'-homology regions to the *URA3* locus, and a bacterial origin of replication and kanamycin marker. The integration cassette was released by Not I digestion and transformed into *S. cerevisiae.* This resulted in genomic integration of potential pantothenate transporter genes at the *URA3* locus.

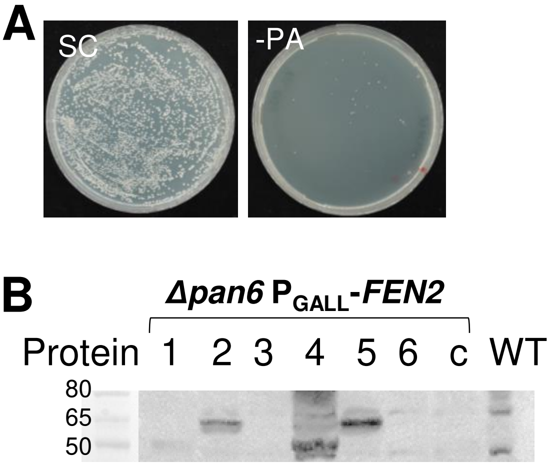

C

	Protein	Expected size (kDa)
1	0A02930	63.57
2	0A05630	67.9
3	0B05620	72.28
4	0D01900	63.03
5	0D03170	74.37
6	0E01180	62.67

Supplementary Figure 4

A. Phenotype of *S. cerevisiae* BY4741 *Δpan6* (Y02304) in SC and SC without pantothenate. **B.** Expression of 3xFLAG-6xHis tagged, potential pantothenate transporters was confirmed by anti-6xHis western blot. 0A02930, 0B05620 and 0E01180 do not seem to be expressed. **C.** Expected sizes of protein products for each tagged pantothenate transporter.
