# Supplementary material for: Pantothenate Auxotrophy in a Naturally Occurring Biocontrol Yeast
Source: Appl Environ Microbiol. 2023 Jul 5;89(7):e00884-23. doi: 10.1128/aem.00884-23 (PMC10370309; doi:10.1128/aem.00884-23)
Supplement: Supplemental file 2 — Supplemental material. Download aem.00884-23-s0002.docx, DOCX file, 0.03 MB [file aem.00884-23-s0002.docx]

**Supplementary Table S1:** Overview of the final *Hanseniaspora meyeri* (APC 12.1) genome assembly.

| **Contigs** | **Chromosomes** | | | | | | | **Mitogenome** |
| --- | --- | --- | --- | --- | --- | --- | --- | --- |
|  | **I** | **II** | **III** | **IV** | **V** | **VI** | **VII** |  |
| **Length [bp]:** | | | | | | | | |
|  | 1,917,773 | 1,513,950 | 1,491,847 | 1,278,704 | 994,218 | 947,899 | 606,242 | 17,078 |
| **Pacbio > 5 kb:** | | | | | | | | |
| Coverage | 104x | 112x | 105x | 102x | 103x | 110x | 102x | 534x |
| Mapped | 99.83 % | | | | | | | |
| **ONT > 20 kb:** | | | | | | | | |
| Coverage | 93x | 117x | 100x | 98x | 97x | 99x | 112x | 160x |
| Mapped | 64.18 % * | | | | | | | |
| **Illumina 2x300 bp:** | | | | | | | | |
| Coverage | 96x | 190x | 101x | 105x | 100x | 110x | 172x | 58x |
| Mapped | 80.49 % ** | | | | | | | |
| **No. of telomere patterns 5’:** | | | | | | | | |
|  | 8 | 19 | 21 | 14 | 1 | 1 | 1 | n.a. |
| **No. of telomere patterns 3’:** | | | | | | | | |
|  | 1 | 16 | 10 | 1 | 2 | 22 | 39 | n.a |
| **Comments:** | | | | | | | | |
| **I, III, IV, V, VI, and VII:** Complete.  **II:** Collapsed rRNA operons at 1055-1075 kb. Two copies present, however, coverage is 10 x higher. The total number of rRNA operons is likely ~20.  **Mitogenome:** Linear, inverted repeats at both ends  *The low number of mapping ONT reads is likely due to the fact that the ONT flow cell was recycled, i.e., it contained reads from a previous run.  ** The lower number of mapping Illumina reads is likely because the genome is diploid and is currently represented in a collapsed haploid version. | | | | | | | | |

**Supplementary Table S2:** Transporter sequences from *S. cerevisiae* S288C and *H. meyeri* APC 12.1 that were used for the analysis shown in Figure 4B. The *S. cerevisiae* sequences were obtained from the Saccharomyces Genome Databes (SGD, [www.yeastgenome.org](http://www.yeastgenome.org)) and the *H. meyeri* sequences from the genome published here.

| **ORF** | **Gene name** | | **Description** |  |
| --- | --- | --- | --- | --- |
| YJR152W | | *DAL5* | Allantoate permease | |
| YAL067C | | *SEO1* | Putative permease | |
| YHL016C | | *DUR3* | Plasma membrane transporter for both urea and polyamines | |
| YCR028C | *FEN2* | | Plasma membrane H^+^-pantothenate symporter |  |
| YGR065C | *VHT1* | | High-affinity plasma membrane H^+^-biotin (vitamin H) symporter |  |
| YLL055W | *YCT1* | | High-affinity cysteine-specific transporter |  |
| YLR004C | *THI73* | | Putative plasma membrane permease |  |
| YGR260W | *TNA1* | | High affinity nicotinic acid plasma membrane permease |  |
| YIL166C | *SOA1* | | Sulfonate and inorganic sulfur transporter |  |
| 0A02930 |  | |  |  |
| 0A05630 |  | |  |  |
| 0B05620 |  | |  |  |
| 0D01900 | *H. meyeri FEN2* | |  |  |
| 0D03170 |  | |  |  |
| 0E01180 |  | |  |  |

**Supplementary Table S3:** Yeasts and filamentous fungi used in this study.

|  | **Isolate** | **SH-number** | **Name** | **Source** | **Features** | **Origin/Reference** |  |
| --- | --- | --- | --- | --- | --- | --- | --- |
| **Yeasts** | | APC 12.1 | SH177122.07FU | *Hanseniaspora meyeri* | Apple bark | Wild type | [10]Switzerland |
|  | | BY4741 |  | *Saccharomyces cerevisiae* |  | MATa his3∆1 leu2∆0 met15∆0  ura3∆0 | Euroscarf |
|  | | Y02304 (PAN6) |  | *Saccharomyces cerevisiae* |  | BY4741 Chr 9 - YIL145c::kanMX4 | Euroscarf |
| **Filamentous fungi** | SHA 18.1 | SH188374.07FU | *Mucor moelleri* | Agricultural soil | Wild type | [10] Switzerland |  |
|  | BC 3.14 | SH231333.06FU | *Penicillium polonicum* | Agricultural soil | Wild type | This work, Switzerland |  |
|  | EC 1.05 | SH177344.07FU | *Botrytis caroliniana* |  | Wild type | [46] Switzerland |  |
|  | Fol4287 |  | *Fusarium oxysporum* f. sp. *lycopersici* |  | Wild type | FGS* |  |

* Fusarium Genetics Stock Center

**Supplementary Table S4:** Primers used in this study for the *FEN2* promoter exchange and to confirm the genomic integrations.

| **Primers** | **Name** | **Sequence** |  |  |  |
| --- | --- | --- | --- | --- | --- |
| **Promoter exchange** | S1-FEN2 | ATTGAGAAGCTGTTTTCAGAATTTTTGATTGGAGAGAGCCTGGGCCTAAGTCATGCGTACGCTGCAGGTGAC | | |  |
|  | S4-FEN2 | AAACAGATTCTCTCTCAACCTCATGTTGAGTGATAGATTTCGATTCCTTCATCATCGATGAATTCTCTGTCG | | |  |
| **Genome Integration** | FEN2 A | GATGATGAAATTTAGCAAAAGAGGA | | |  |
|  | FEN2 B | AATTATTGGGGACCATACCTACAAT |  |  |  |
|  | URA3 3`f | AGAGCACTTGAATCCACTGC |  |  |  |
|  | URA3 3’r | GATTTGGTTAGATTAGATATGGTTTC |  |  |  |
|  | URA3 5’f | GGGCGGATTACTACCGTT |  |  |  |
|  | URA3 5’r | GTAATGTTATCCATGTGGGC |  |  |  |

**Supplementary Table S5:** Genome sequences used for the phylogenetic analysis. The *H. meyeri* isolate APC 12.1 reported in this study is shaded in blue.

| **Name** | **Isolate** | | **Accession Nr.** | |
| --- | --- | --- | --- | --- |
| *H. clermontiae* | | NRRL Y27515 | GCA_003706385.1 | |
| *H. gamundiae* | | CRUB 1928 | GCA_003020785.1 | |
| *H. guilliermondii* | | UTAD 222 | GCA_900119595.1 | |
| *H. guilliermondii* | NRRL Y-1625 | | GCA_004919775.1 | |
| *H. hatyaiensis* | ZIM 2327 | | GCA_003708765.1 | |
| *H. jakobsenii* | ZIM 2603 | | GCA_004919785.1 | |
| *H. lachancei* | NRRL Y-27514 | | GCA_004919765.1 | |
| *H. lindneri* | CBS 285 | | GCA_019649525.1 | |
| *H. meyeri* | NRRL Y-27513 | | GCA_004919825.1 | |
| *H. meyeri* | APC 12.1 | |  | |
| *H. mollemarum* | CBS 15034 | | GCA_019649515.1 | |
| *H. nectarophila* | CBS 13382 | | GCA_004919845.1 | |
| *H. occidentalis* | BCS 6783 | | GCA_004919885.1 | |
| *H. occidentalis* | NRRL Y-7946 | | GCA_004919895.1 | |
| *H. opuntiae* | AWRI 3578 | | GCA_001749795.1 | |
| *H. osmophila* | AWRI 3579 | | GCA_001747045.1 | |
| *H. osmophila* | NRRL Y-1613 | | GCA_003707715.1 | |
| *H. pseudoguillermondii* | ZIM 213 | | GCA_003708335.1 | |
| *H. singularis* | ZIM 2326 | | GCA_003708365.1 | |
| *Hanseniaspora* sp. | CRUB 1602 | | GCA_004919795.1 | |
| *H. uvarum* | 34-9 | | GCA_000775265.3 | |
| *H. uvarum* | AWR 13580 | | GCA_001747055.1 | |
| *H. uvarum* | DSM 2768 | | GCA_000968475.1 | |
| *H. valbyensis* | Y-1626 | | GCA_001664025.1 | |
| *H. vineae* | T02/19AF | | GCA_000585475.3 | |
| *H. vineae* | Y-17529 | | GCA_004919945.1 | |
| *S. cerevisiae* | S288C | | GCA_000146045.2 | |
